# Nutrient Availability as a Mechanism for Selection of Antibiotic Tolerant *Pseudomonas aeruginosa* within the CF Airway

**DOI:** 10.1371/journal.ppat.1000712

**Published:** 2010-01-08

**Authors:** Lucas R. Hoffman, Anthony R. Richardson, Laura S. Houston, Hemantha D. Kulasekara, Willm Martens-Habbena, Mikkel Klausen, Jane L. Burns, David A. Stahl, Daniel J. Hassett, Ferric C. Fang, Samuel I. Miller

**Affiliations:** 1 Department of Pediatrics, University of Washington, Seattle, Washington, United States of America; 2 Department of Microbiology, University of Washington, Seattle, Washington, United States of America; 3 Department of Genome Sciences, University of Washington, Seattle, Washington, United States of America; 4 Department of Civil and Environmental Engineering, University of Washington, Seattle, Washington, United States of America; 5 Department of Molecular Genetics, Biochemistry and Microbiology, University of Cincinnati College of Medicine, Cincinnati, Ohio, United States of America; 6 Department of Medicine, University of Washington, Seattle, Washington, United States of America; 7 Department of Laboratory Medicine, University of Washington, Seattle, Washington, United States of America; Stanford University School of Medicine, United States of America

## Abstract

Microbes are subjected to selective pressures during chronic infections of host tissues. *Pseudomonas aeruginosa* isolates with inactivating mutations in the transcriptional regulator LasR are frequently selected within the airways of people with cystic fibrosis (CF), and infection with these isolates has been associated with poorer lung function outcomes. The mechanisms underlying selection for *lasR* mutation are unknown but have been postulated to involve the abundance of specific nutrients within CF airway secretions. We characterized *lasR* mutant *P. aeruginosa* strains and isolates to identify conditions found in CF airways that select for growth of *lasR* mutants. Relative to wild-type *P. aeruginosa*, *lasR* mutants exhibited a dramatic metabolic shift, including decreased oxygen consumption and increased nitrate utilization, that is predicted to confer increased fitness within the nutrient conditions known to occur in CF airways. This metabolic shift exhibited by *lasR* mutants conferred resistance to two antibiotics used frequently in CF care, tobramycin and ciprofloxacin, even under oxygen-dependent growth conditions, yet selection for these mutants *in vitro* did not require preceding antibiotic exposure. The selection for loss of LasR function *in vivo*, and the associated adverse clinical impact, could be due to increased bacterial growth in the oxygen-poor and nitrate-rich CF airway, and from the resulting resistance to therapeutic antibiotics. The metabolic similarities among diverse chronic infection-adapted bacteria suggest a common mode of adaptation and antibiotic resistance during chronic infection that is primarily driven by bacterial metabolic shifts in response to nutrient availability within host tissues.

## Author Summary

Chronic infections are distinguished from many other infections in that they are difficult to eradicate with antibiotics. Thus, the microbes that cause chronic infections persist within host tissues for long periods despite our best treatment efforts. During the course of these chronic infections, the causative microbes often change genetically. For example, a bacterium that commonly infects the lungs of people with the genetic disease cystic fibrosis (CF) undergoes several known changes that affect the growth of this pathogen. However, the causes and clinical impact of the changes undergone by this and other chronically infecting microbes are unclear. We show that a common, early mutation found in bacteria isolated from chronically infected CF airways renders these bacteria better able to grow in the nutrients found in CF lung secretions. Interestingly, these same changes also confer resistance to several antibiotics used commonly to treat CF patients. Many of the characteristics conferred by this mutation are exhibited by other microbes found in chronic infections, suggesting that adaptation of these microbes to host tissue nutrient environments may be a common mechanism of antibiotic resistance in chronic infections.

## Introduction

Microbes are subjected to selection in host environments during the course of chronic infections [1],[2],[3]. The characteristics selected may have profound impacts on disease outcomes, particularly if they confer increased microbial fitness or resistance to therapy. One example of this phenomenon is the adaptation of *Pseudomonas aeruginosa* within the airways of people with cystic fibrosis (CF). Diverse phenotypic changes have been observed among CF chronic *P. aeruginosa* infection isolates, including changes in several surface antigens [4],[5], altered antibiotic susceptibilities [6], and overproduction of the mucoid exopolysaccharide alginate [3]. *P. aeruginosa* CF adaptive changes have been associated with poor clinical outcomes [7],[8] and, in the case of mucoidy, a diminished likelihood of eradication by antibiotics [9].

Recently, several groups have described *P. aeruginosa* CF isolates with inactivating mutations in the gene *lasR* [2],[8],[10],[11],[12]. Genetic analyses demonstrated that these mutants emerged from existing, chronically-infecting lineages, as opposed to representing new infections, and that multiple lineages with independent *lasR* mutations occurred within individual patients, indicative of strong selective pressure against LasR function [2],[11]. *lasR* encodes a central regulator of the bacterial intercellular signaling system known as quorum sensing that requires the synthesis and recognition of *P. aeruginosa* small molecule products, including acyl-homoserine lactones (AHL). *lasR* mutant isolates occur in at least one-third of *P. aeruginosa* culture-positive individuals younger than 15 years attending CF clinics in Seattle [2],[8]. Among this population, *lasR* mutant isolates emerged relatively early during CF airway infection (on average 2 years before mucoidy), and were associated with worse lung function [8]. LasR inactivation conferred distinct phenotypic consequences, including distinctive colony morphology (autolysis and surface iridescent sheen) that facilitates the identification of mutant isolates, a growth advantage in specific amino acids abundant in CF secretions [13], and increased β-lactamase enzyme activity [2],[11]. These growth phenotypes suggested that selection may be due to exposure to antibiotics and nutrient availability within the CF airway. The latter possibility was further indicated by altered growth in specific nitrogen sources by *lasR* mutants compared with their wild-type counterparts [11].

**Figure 1:**
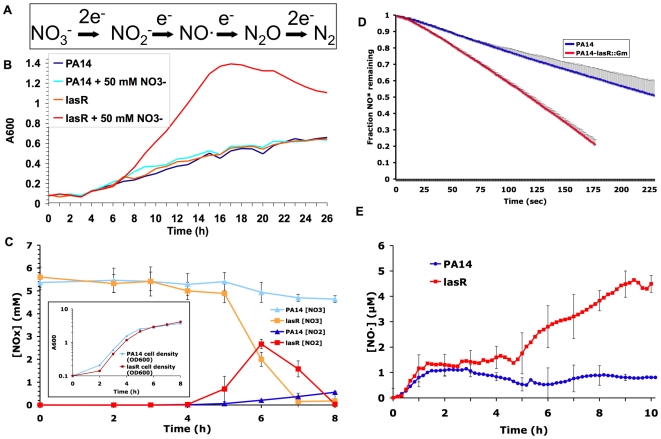
Mutation in *lasR* increases denitrification, and leads to the buildup of the toxic metabolite NO·. (A) The reactions that comprise the dissimilatory denitrification pathway, or the serial reduction of NO_3_^−^ to nitrogen gas, in bacteria. (B) Growth of the wild-type *P. aeruginosa* strain PA14 and its derived *lasR* mutant PA14-*lasR*::Gm in a chemically defined medium (PN minimal medium) with and without NO_3_^−^ supplementation, under low oxygen mass-transfer conditions. Results representative of three separate experiments. (C) Nitrate (NO_3_^−^) and nitrite (NO_2_^−^) concentrations in shaken cultures of the indicated strains grown with LB and 5 mM added nitrate. Results shown are the averages of technical triplicates ±s.d. and are representative of two separate experiments. Slopes for lines fit to each dataset between 4 and 6 minutes were significantly different with p<0.001 for [NO_2_^−^] and p<0.02 for [NO_3_^−^]. Also shown for reference (inset) are concurrent growth curves for each strain; results representative of two separate experiments. (D) Rates of degradation of added NO· from a mixture of NO· donors (DEANO and ProliNO) by cells pre-grown in a sealed chamber in LB prior to donor addition. Results shown are average ±s.d. for technical duplicates and are representative of two separate experiments. Slopes for lines fit to each dataset between 25 and 100 seconds were significantly different (p<0.02). (E) NO· concentrations in sealed, stirred LB cultures of the indicated strains during growth with 50 mM added NO_3_^−^. Results are average ±s.d. of three separate experiments. Slopes for lines fit to each dataset between 4 and 9 minutes were significantly different between wild-type and *lasR* mutant cultures (p<0.02). For all experiments shown, similar results were obtained in at least two separate experiments with the Patient 1 early isolate and its derived *lasR* mutant (described in Table 1), and complementation with the *lasR* gene on a plasmid restored wild-type phenotypes (not shown).

AHL signaling was shown previously by transcriptional microarray [14] and enzymatic analyses [15],[16] to regulate the *P. aeruginosa* nitrogen metabolic pathway known as denitrification (Fig. 1A). However, the *las* system comprises only a portion of the complex AHL regulon, and the metabolic consequences of LasR inactivation were not defined previously. Previous evidence from a global physiological analysis of clinical isolates indicated that *lasR* mutation could confer a growth advantage in the denitrification substrates nitrate (NO_3_^−^) and nitrite (NO_2_^−^) [11], suggesting that *lasR* mutant *P. aeruginosa* cells may exhibit increased utilization of NO_3_^−^ and NO_2_^−^ as electron acceptors. Conversely, LasR inactivation conferred sensitivity to high concentrations of NO_2_^−^ among these isolates [11], as would be predicted if *lasR* mutant cells avidly metabolize NO_2_^−^ to nitric oxide (NO·), the chief toxic metabolic side-product of denitrification (Fig. 1A).

The airways of people with CF are known to contain abundant concentrations of NO_3_^−^ and other nitrogen species [17],[18], while the concentrations of NO· (an important antimicrobial component of host innate immunity) are usually significantly lower than in people without CF for as yet unknown reasons [19]. In addition, CF secretions infected with *P. aeruginosa* include areas with very low molecular oxygen tensions [20]. These conditions would tend to favor the use among infecting microbes of nitrogen oxides as electron acceptors at the expense of oxygen utilization [16],[21]. Thus, we hypothesized that *lasR* mutant *P. aeruginosa* cells have respiratory alterations favoring growth in the nitrogen and oxygen conditions characteristic of CF airways. Since many antibiotics work best under aerobic conditions [22],[23], such a metabolic shift could adversely affect susceptibility (and thus clinical response) to antibiotics. To test these hypotheses, we defined the consequences of *lasR* mutation with respect to nitrate and oxygen metabolism, as well as antibiotic susceptibility, in laboratory strains and CF clinical isolates of *P. aeruginosa*.

## Results

### *lasR* mutants have a growth advantage in NO_3_^−^ and accumulate the toxic metabolite nitric oxide (NO·)

Given the evidence that *lasR* mutants may have a growth advantage in NO_3_^−^ [11], we compared the growth of *P. aeruginosa* laboratory strain PA14 and clinical isolates carrying wild-type *lasR* alleles with their derived *lasR* mutant strains in the presence of various concentrations of NO_3_^−^. *lasR* mutants exhibited a substantial growth advantage in minimal medium with added NO_3_^−^. As shown in Fig. 1B for a *lasR* mutant with a gentamicin insertion cassette derived from PA14 (PA14-*lasR*::Gm), a growth advantage was detected in NO_3_^−^ concentrations as low as 125 µM, well below the average NO_3_^−^ concentrations recently measured in CF airway secretions [17],[18], and the advantage was more pronounced at higher NO_3_^−^ concentrations (not shown). The average rate of *lasR* mutant growth (calculated as the slopes of lines fit to the datasets shown between 8 and 16 minutes) in 50 mM NO_3_^−^ was increased ∼5-fold relative to wild-type. Similar results were obtained using Luria Broth, with PA14 with an unmarked *lasR* deletion (PA14Δ*lasR*), and with paired *lasR* wild-type and mutant clinical isolates (not shown). This analysis confirms and extends our previous finding that *lasR* mutations confer a growth advantage with nitrogen sources that are abundant in the CF airway, including NO_3_^−^, as well as with aromatic amino acids [11].

*lasR* mutant strains and isolates converted NO_3_^−^ to NO_2_^−^, and degraded NO_2_^−^, at significantly higher rates than did wild-type strains and isolates (Fig. 1C). For example, the average rate of NO_2_^−^ production by *lasR* mutant strains was ∼4.4-fold greater than by wild-type. In contrast, *lasR* mutant strains and isolates demonstrated a relatively modest spontaneous increase in NO· reduction relative to wild-type (Fig. 1D); slopes for lines fit to each dataset in Fig. 1D between 25 and 100 seconds demonstrated that *lasR* mutant cells had an NO· degradation rate only ∼1.8-fold greater than wild-type cells. These activities resulted in dramatically higher levels of NO· (Fig. 1E) in *lasR* mutant cultures that could not be explained by any concurrent difference in growth rates between *lasR* mutants and wild-type in added NO_3_^−^ (compare Figs. 1B and 1E). The accumulation of NO·, a potent microbicide [24], in *lasR* mutant cultures would be predicted to result in cell death at very high cell densities (as observed with *P. aeruginosa* cells with mutations in the quorum sensing regulator *rhlR* [16]) and in increased susceptibility to exogenous NO· sources.

### *lasR* mutation confers increased susceptibility to nitrosative stress, and selection requires a membrane-bound NO_3_^−^ reductase

**Figure 2:**
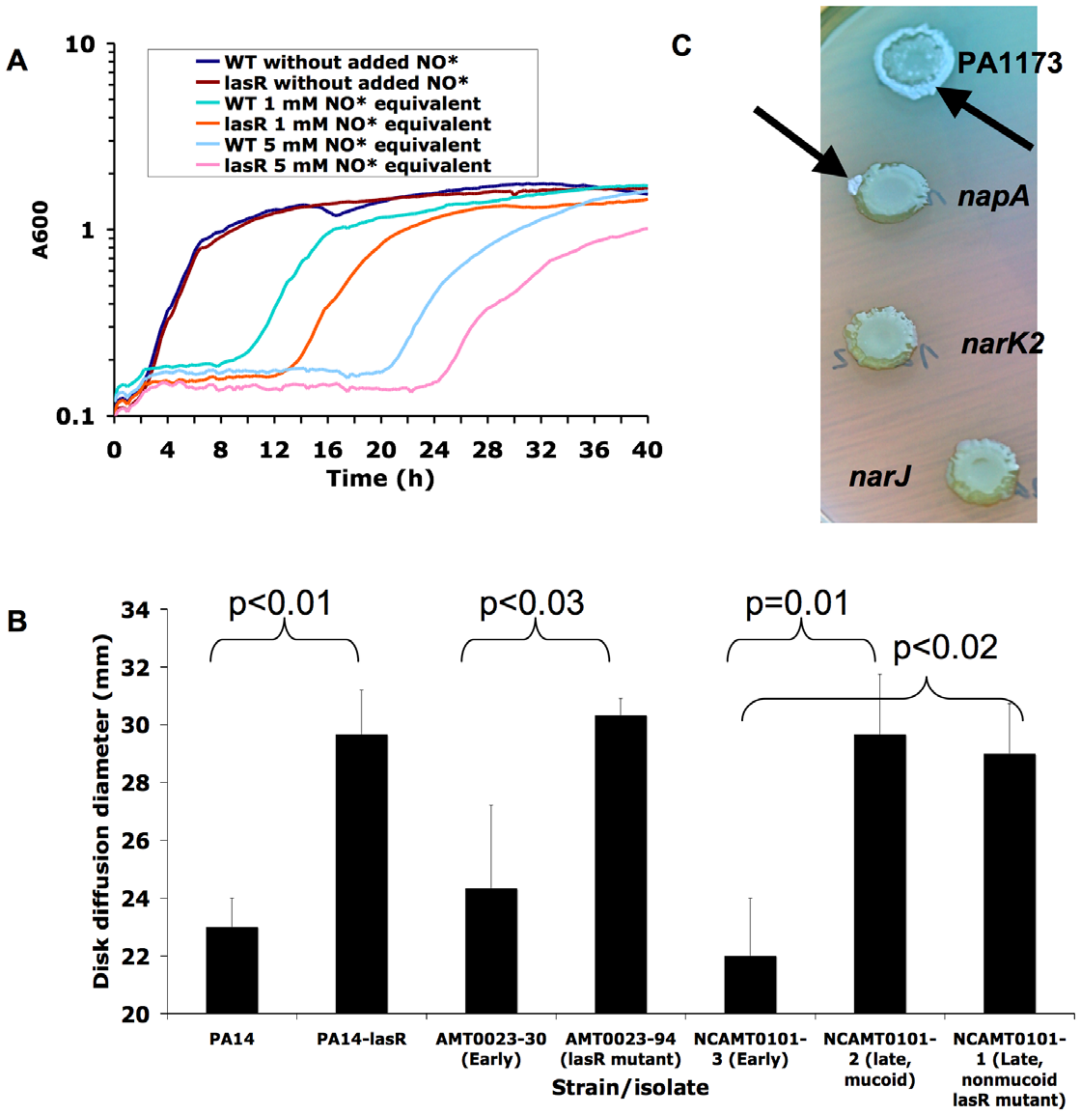
Mutation in *lasR* confers increased susceptibility to nitrosative stress, including acidified NO_2_^−^. (A) Growth rate of PA14 versus PA14-*lasR*::Gm in LB in the presence and absence of two amounts of added NO· donor SPER-NO, transiently generating the indicated concentrations of NO·. Each result representative of at least two separate experiments. (B) Disk diffusion diameters of the indicated strains and isolates on LB agar buffered to pH 6.5, with disks containing 100 µmol of NaNO_2_, then incubated for 24 hours at 37°C under aerobic conditions. Average ±s.d. for triplicate experiments. Similar results were obtained with PA14Δ*lasR*, and complementation with a wild-type copy of *lasR* on a plasmid restored wild-type phenotypes to *lasR* mutants (not shown). (C) Spontaneous sectors displaying the *lasR* phenotype (metallic surface sheen and autolytic flattening, indicated by black arrows) arise during agar surface growth for 1 week of PA14-derived strains with transposon insertions in genes in the periplasmic NO_3_^−^ reductase gene cluster (top, genes PA1173 and *napA*) but not from those with insertion in genes in the membrane-bound NO_3_^−^ reductase gene cluster (bottom, genes *narJ* and *narK2*). Also visible around the *lasR* mutant sectors is the blue pigment pyocyanin, which is produced at higher levels by *lasR* mutant PA14 than by wild-type cells upon extended incubation [61]. Results representative of four separate experiments. Complementation with a wild-type copy of *lasR* on a plasmid restored wild-type phenotypes to *lasR* mutants isolated from sectors (not shown).

Because *lasR* mutant *P. aeruginosa* produces elevated levels of endogenous NO·, and bacterial cells possess a finite capacity for detoxifying NO· that can be exceeded by exposure to exogenous reactive nitrogen species (RNS) [25], we predicted that *lasR* mutants would also be more susceptible to the exogenous nitrosative stress presented by either NO· donors (which have relatively short aqueous half-lives [26]) or acidified NO_2_^−^ (with substantially greater aqueous half-life [27]). Therefore, *lasR* mutant strains and isolates were tested for these susceptibility phenotypes. *lasR* mutants were more susceptible to growth inhibition by the addition of NO· donor compounds to liquid cultures (Fig. 2A), and by NO_2_^−^ disks during growth on acidified agar medium (Fig. 2B). These results are in agreement with our previous phenotype array findings that *lasR* inactivation in clinical *P. aeruginosa* isolates confers increased susceptibility to high concentrations of NO_2_^−^ in unbuffered liquid minimal medium [11]. Furthermore, analysis of clinical isolate pairs demonstrated that the impact of *lasR* mutation on NO_2_^−^ susceptibility was similar to the effect demonstrated previously for mucoidy [27], as shown in Fig. 2B for one isolate pair (NCAMT0101-2 and -3).

*P. aeruginosa* encodes two NO_3_^−^ reductases, one in the bacterial inner membrane and the other in the periplasm. It was found previously that, of these two, only the membrane-bound enzyme was required for anaerobic growth of *P. aeruginosa* [17]. Interestingly, we found that spontaneous *lasR* mutants did not emerge during extended growth on agar medium from strains with transposon insertions in genes encoding subunits of the membrane-bound NO_3_^−^ reductase (*narJ* and *narK2*), while sectors displaying the characteristic *lasR* phenotype arose frequently among strains with similar mutations in genes encoding the periplasmic enzyme (PA1173 and *napA*) (Fig. 2C). Furthermore, the growth advantage in NO_3_^−^ conferred by *lasR* mutation (Fig. 1B) was not observed in the absence of *narK* genes (*narK1narK2lasR*, data not shown). Thus, the membrane-bound NO_3_^−^ reductase was required for both the growth advantage of *lasR* mutants in added NO_3_^−^ and for rapid *lasR* mutant emergence *in vitro*. These results functionally link the growth advantage in NO_3_^−^ conferred by a *lasR* mutation with the selection of these mutants, at least *in vitro*, and perhaps also in the NO_3_^−^ -rich CF airway [17],[18].

### Factors that detoxify NO· increase *P. aeruginosa lasR* mutant growth

**Figure 3:**
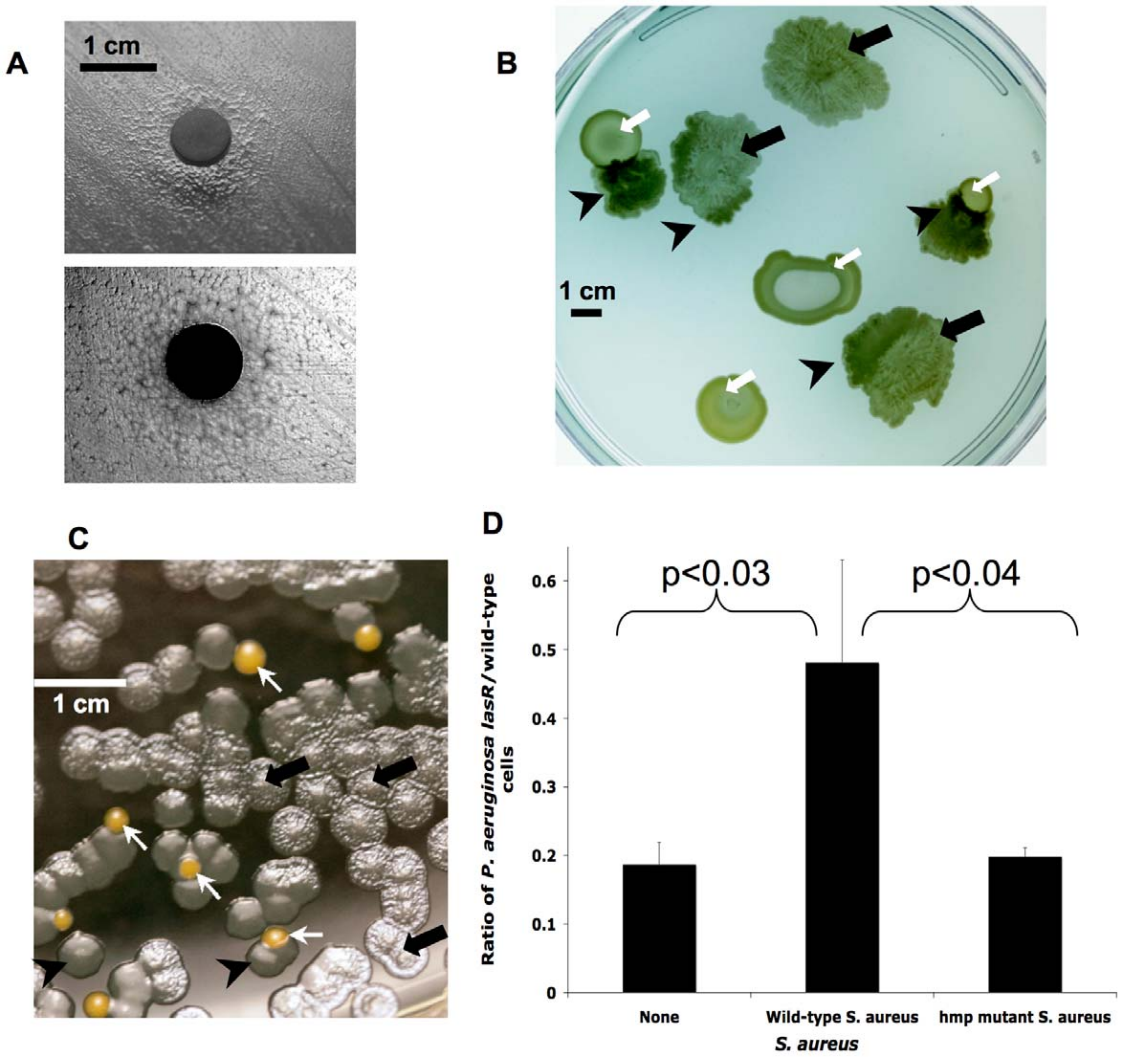
*lasR* mutant *P. aeruginosa* growth is altered during co-culture with *S. aureus*, apparently due to detoxification of NO·. (A) Cells in a lawn of *lasR* mutant PA14 growing near a disk containing hemoglobin, which stoichiometrically scavenges NO· (as opposed to the catalytic effect of *S. aureus* colonies in B–C), do not display the autolysis of cells more distant from the disk, as shown in a photograph from above with illumination from above (upper) and below (lower). (B) Clinical isolates of *lasR* mutant *P. aeruginosa* (black arrows) and *S. aureus* (white arrows) grown together on LB agar with 400 µM added KNO_3_, with *lasR* colony autolysis and resulting translucency indicated through transillumination of the agar plate. Black arrows indicate areas of *P. aeruginosa* lysis and/or sheen, white arrows indicate colonies of *S. aureus*, and black arrowheads indicate where *lasR* colony autolysis is relieved in the presence of *S. aureus*. (C) Co-culture as in (B) except with laboratory strain PA14-*lasR::*Gm grown with wild-type *S. aureus* Newman strain (colored orange *in silico* for clarity) after inoculation at a cell ratio of 50∶1. Arrows as in (B). (D) Ratios of cell counts of *P. aeruginosa lasR* versus wild-type after inoculation of static cultures in liquid LB with 400 µM added KNO_3_ with equal numbers of each *P. aeruginosa* strain followed by growth for 48 hours in the presence and absence of equal cell numbers of the indicated *S. aureus* strains. Results are averages ±s.d. for triplicate counts and are representative of three separate experiments. Total final cell count was similar in each experiment.

The findings that *lasR* mutants overproduce the potent microbicide NO· (Fig. 1E), that they undergo autolysis at high cell density [11], and that *lasR* mutants exhibit increased growth inhibition by exogenous sources of NO· (Fig. 2A) suggested that factors that detoxify NO· could enrich for *lasR* mutant growth. This hypothesis is supported by the observation that the cell death observed when RhlR mutants are grown anaerobically as biofilms can be prevented by addition of an NO· scavenger [16]. Similarly, we found that *P. aeruginosa lasR* mutants growing near disks containing hemoglobin, which scavenges NO· stoichiometrically, also grew better than did cells farther away from the disk (Fig. 3A). These results suggest that the presence of an NO· “sink” such as hemoglobin increased growth of *lasR* mutant *P. aeruginosa*.

Some bacteria, including the gram-positive CF bacterial pathogen *Staphylococcus aureus*, are known to be relatively resistant to the effects of NO· as a result of efficient cellular detoxification mechanisms [24]. Furthermore, we found previously that the presence of live, but not dead, *S. aureus* decreased expression of a *P. aeruginosa* gene (*fhp* [25]) involved in NO· degradation [28], suggesting that *S. aureus* may detoxify NO· produced by *P. aeruginosa*. The catalytic effect of growing *S. aureus* cells would be predicted to be even more robust than that of the stoichiometric agent hemoglobin. Therefore, we compared the growth of *lasR* mutants and wild type bacteria in the presence and absence of *S. aureus*.

### The CF pathogen *S. aureus* increases the growth of *P. aeruginosa lasR* mutants, apparently through NO· detoxification

When grown near *S. aureus*, *lasR* mutants exhibited wild-type growth phenotypes, as manifested by thicker colonies, using either clinical isolates or laboratory strains of each species (Figs. 3B–C). This phenotypic change did not require contact with *S. aureus*. Cell-free culture medium, cell sonicates, and organic extracts of *S. aureus* cultures did not exhibit the activity of *S. aureus* colonies, suggesting that *S. aureus* cell activity was required for this phenotypic change.

To further characterize the growth of *lasR* mutants and its modification by *S. aureus*, we inoculated static, liquid cultures with equal numbers of *P. aeruginosa* wild-type and *lasR* mutant cells in the presence or absence of wild-type or mutant *S. aureus* partially defective for NO· degradation (*hmp* mutants [24]), and measured the growth of each strain after incubation. As in previous experiments (e.g., Fig. 2A) [11], *P. aeruginosa lasR* mutants grown alone did not have a growth defect relative to wild-type strains and isolates in these nutrient conditions (not shown). We found that *lasR* mutant growth was enhanced by co-culture with wild-type *S. aureus*, but not by *hmp* mutant *S. aureus* (Fig. 3D). In addition, *lasR* mutant colonies growing on LB agar near colonies of *hmp* mutant *S. aureus* displayed substantially more autolysis than did *lasR* mutants growing near wild-type *S. aureus* (not shown), supporting the notion that *S. aureus* NO· detoxification is required to impede *lasR P. aeruginosa* colony autolysis. These results suggest that the presence of *S. aureus*, which commonly co-infects CF airways with *P. aeruginosa* [29], encourages the growth of *lasR* mutant *P. aeruginosa* by detoxifying NO·. This effect of *S. aureus* and other microbes could contribute to the relatively low tensions of NO· observed within CF airways [19], which would be predicted to further encourage the growth of *lasR* mutant *P. aeruginosa* by providing a mechanism to mitigate the toxic effects resulting from the shift to nitrate metabolism.

### Oxygen utilization is diminished in *lasR* mutants, resulting in resistance to oxidative stress

**Figure 4:**
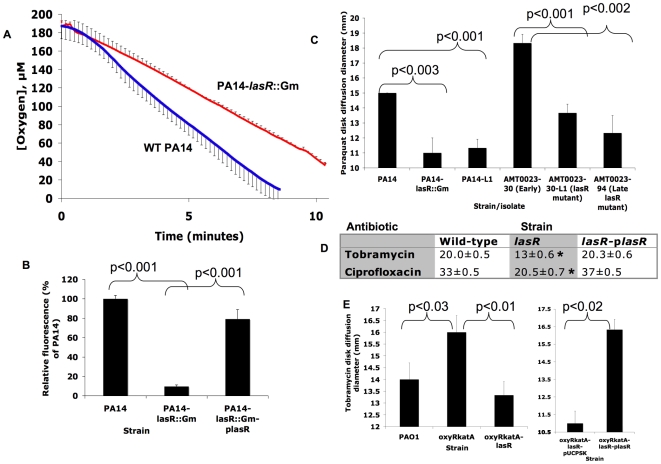
*lasR* mutant *P. aeruginosa* strains and isolates exhibit lower rates of oxygen utilization and resistance to paraquat, tobramycin and ciprofloxacin. (A) Change in oxygen concentration during stirred incubation of washed cells of the indicated strains resuspended at equivalent cell densities in LB with 400 µM KNO_3_ at 37°C. Average of 3 experiments ±s.d.; results representative of 3 separate experiments. Slopes for lines fit to each dataset between 1 and 5 minutes were significantly different (p<0.04). Complementation of *lasR* mutants with a wild-type copy of *lasR* on a plasmid restored wild-type phenotypes (not shown). The difference was no longer statistically significant in the absence of added KNO_3_ (LB was shown previously to contain approximately 23 µM NO_3_^−^ [71]; not shown). (B) Fluorescence yields generated by adding a saturated DMSO solution of hydroethidine (HE), a probe of superoxide concentration [32], for 5 minutes on lawns of the indicated strains (where p*lasR* indicates complementation with a wild-type copy of *lasR* on a plasmid) grown on LB agar. Average ±s.d. of triplicates and representative of five separate experiments; similar results were obtained in liquid cultures and with clinical isolate pairs for Patient 1 (not shown). (C) Zone diameters of growth inhibition for the indicated clinical isolates and strains by disks containing 1 µmol of paraquat after 24 hours' incubation in air at 37°C on LB agar with 400 µM KNO_3_. Results shown are average ±s.d. for triplicates and are representative of >10 separate experiments. Complementation with a copy of *lasR* on a plasmid restored wild-type phenotypes to *lasR* mutants (data not shown). (D) As in (C), except with disks containing 3.75 µg of ciprofloxacin or 3 µg of tobramycin on MH agar and 400 µM KNO_3_ (the *lasR* mutant strain tested for tobramycin susceptibility was PA14-L1, which does not contain an engineered aminoglycoside resistance gene). Average ±s.d. for triplicates. *, p<0.001 compared both with wild-type and the complemented mutant. No decreases in susceptibility were noted with disks of control antibiotics: carbenicillin, tetracycline, aztreonam, and polymyxin. Results with the unmarked deletion strain PA14Δ*lasR* were similar to those with the *lasR* mutants shown for both (C) and (D). (E) Tobramycin disk diffusion diameters for experiments as in 5d except with the indicated strains. Experiment at right compares the *oxyRkatA-lasR* mutant carrying an empty plasmid vector with the same strain carrying the same plasmid but with a wild-type copy of *lasR*, and on agar media containing 300 µg/mL carbenicillin for plasmid maintenance. Similar results were observed for disks of ciprofloxacin (not shown). Results shown are averages ±s.d. for triplicates.

Low molecular oxygen tension and abundant nitrogen oxides have been observed in CF secretions [18],[20]. Furthermore, deficiency in *las* signaling has been shown to result in decreased expression of cytochromes central to oxygen utilization [30]. Therefore, *P. aeruginosa lasR* mutants could have decreased utilization of oxygen as an electron acceptor. To test this hypothesis, we examined rates of oxygen utilization in liquid (Fig. 4A) and agar-grown (not shown) *P. aeruginosa* cultures. *lasR* mutant cultures exhibited oxygen consumption rates at approximately 40–50% those of wild-type cultures (determined by comparing slopes of lines fit to each dataset from 1–5 minutes in Fig. 4A). Aerobic metabolism generates toxic reactive oxygen species (ROS), including superoxide (O_2_^−^·) [31]. As *lasR* mutant cells exhibit decreased rates of oxygen utilization relative to wild-type cells (Fig. 4A), *lasR* mutant cells could consequently contain lower endogenous levels of ROS. Hydroethidine is a specific fluorescent indicator of intracellular O_2_^−^· [32]. Hydroethidine addition to air-grown agar (Fig. 4B) or liquid (not shown) cultures of *lasR* mutants yielded much lower cell fluorescence than did its addition to wild-type cultures. We demonstrated that cell permeability was equivalent in wild-type PA14 and *lasR* mutant cells using two established methods: one measuring uptake of ethidium bromide during efflux pump chemical blockade [33] and another based on the uptake of the fluorescent molecule NPN without efflux pump inactivation [34] (data not shown). These results indicate that intracellular O_2_^−^· concentrations are lower in *lasR* mutant cells than in wild-type cells. By analogy to the increased susceptibility of *lasR* mutants to exogenous nitrosative stress associated with higher endogenous NO· production (Figs. 1E and 2A–B), these results suggest that *lasR* mutant cells would be more resistant to exogenous sources of oxidative stress, including redox-cycling agents [31],[35].

To test this hypothesis, we measured the response of *P. aeruginosa* cultures to the redox-cycling agent paraquat, which reacts with intracellular oxygen to generate O_2_^−^· [35]. As shown in Fig. 4C, cultures of *lasR* mutants (in both laboratory strain and clinical isolate backgrounds) were more resistant to paraquat and, as with nitrite susceptibility (Fig. 2B, far right), this effect was present in *lasR* mutant clinical isolates after several years of infection (Fig. 4C, far right). Differences in susceptibility to exogenous hydrogen peroxide exhibited the same trend, but to a lesser extent (not shown). Thus, the susceptibility of *lasR* mutant *P. aeruginosa* to exogenous oxidative stress is altered, apparently due to lower endogenous production of ROS and higher residual capacity for detoxification. Polyacrylamide gel enzymatic activity assays [31] demonstrated that *lasR* mutant cells and their wild-type counterparts exhibited similar activities of superoxide dismutases, enzymes that degrade O_2_^−^· (data not shown), supporting the concept that the differences in endogenous O_2_^−^· levels, and susceptibility to paraquat, were due to differences in O2^−^· production rather than differences in O_2_^−^· degradation.

### *lasR* mutants have a growth advantage under conditions of oxidative stress

**Figure 5:**
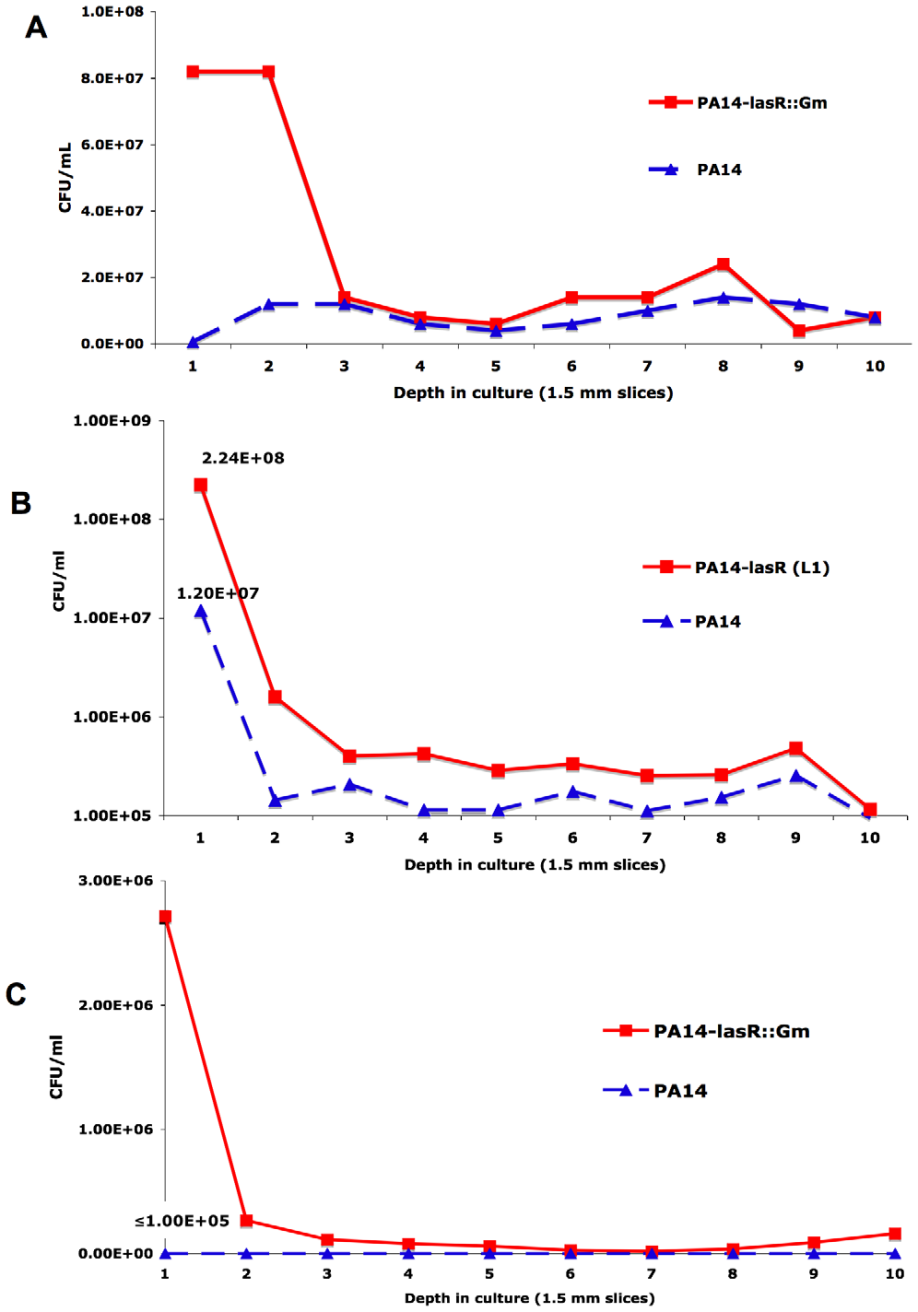
Resistance of *lasR* mutant *P. aeruginosa* to tobramycin and ciprofloxacin is oxygen-dependent. (A) Colony counts from serial slices of an agar-suspended culture with LB+400 µM KNO_3_ containing 5 mM paraquat inoculated with equal numbers of cells of PA14 and a derived *lasR* mutant (PA14-*lasR*::Gm) and incubated for 48 hours. (B) The same experiment as in (A), except with 1 µg/mL tobramycin instead of paraquat, and using PA14-L1 (because this *lasR* mutant lacks an aminoglycoside resistance cassette). (C) The same experiment as in (B), except with 0.25 µg/mL ciprofloxacin and with PA14-*lasR*::Gm. All results representative of at least 3 independent experiments. No differences in cell density were noted in the absence of antibiotics or paraquat under these conditions after 48 hours of growth (not shown), in agreement with liquid growth findings [11].

The lower endogenous O_2_^−^· concentrations of *lasR* mutants indicate that they might have a growth advantage compared with wild-type cells when grown under conditions of oxidative stress. To test this hypothesis, agar-suspended cultures were inoculated with equal numbers of *lasR* and wild-type cells in the presence of paraquat, and then the density of each strain was determined in serial, thin culture slices. Using an oxygen microprobe, oxygen concentration within these cultures became undetectable within approximately 2 mm of depth below the surface after 24 hours of incubation (data not shown). This growth medium is a viscous gel, limiting the motility and sedimentation of cells and thus preserving two-dimensional culture structure, resulting in the establishment of a stable oxygen gradient. In this way, this culture may reproduce some aspects of CF respiratory secretions, which are relatively viscous compared to liquid cultures and exhibit oxygen gradients [20]. Furthermore, as ROS such as O_2_^−^· are side-products of oxygen-based respiration, ROS are produced at decreasing amounts with increased depth within the cultures. As shown in Fig. 5A, *lasR* cells greatly outcompeted wild-type cells at more superficial depths, where oxygen was detectable and O_2_^−^· could be produced upon paraquat exposure. This effect diminished with increasing depth and, therefore, with lower oxygen concentration. Thus, under growth conditions in which superoxide is generated, *lasR* mutants have a relative fitness advantage.

### The shift to increased nitrate based metabolism by *lasR* mutants confers tolerance to antibiotics commonly used in CF treatment

One condition under which ROS are generated within bacterial cells is upon exposure to bactericidal antibiotics, including fluoroquinolones and aminoglycosides, under aerobic conditions [22],[36]. Bacterial killing by both classes of antibiotics has been shown to be attributable in part to induction of superoxide production [37]. In addition, efficient aminoglycoside uptake (and thus bacterial killing) requires aerobic electron transport [23]. Furthermore, a *las*-regulated *P. aeruginosa* exoproduct, the *Pseudomonas* quinolone signal, induces an oxidative stress response, increased cellular ROS, and increased susceptibility to fluoroquinolones in *P. aeruginosa* [38], functionally linking response to oxidative stress and susceptibility to fluoroquinolones. A relationship between fluoroquinolone susceptibility and oxidative stress is supported by work in other bacterial species [39], including the observation that spontaneous mutants in superoxide response regulators have been selected by exposure of both *Escherichia coli* and *Salmonella enteritidis* to fluoroquinolones [40]. Thus, we predicted that the lower oxygen utilization rates and increased resistance to sources of superoxide exhibited by *lasR* mutants would result in decreased susceptibility to the fluoroquinolone ciprofloxacin and the aminoglycoside tobramycin, both of which are used frequently to treat CF patients [41]. As shown in Fig. 4D, surface cultures on nitrate-containing agar medium of *lasR* mutants were less susceptible to disks containing these drugs. Agar-suspended cultures in the same medium demonstrated that these differences were oxygen-dependent (Figs. 5B–C), as with paraquat (Fig. 5A). These results suggest that, under these culture conditions, inactivating *lasR* mutation confers resistance to two of the antibiotics used most frequently in CF care, tobramycin and ciprofloxacin. To further investigate the relationship between oxidative stress and antibiotic resistance, we compared the susceptibilities to oxidative stress and antibiotics of strains of *P. aeruginosa* carrying the double mutation *oxyRkatA*, or the triple *mutation oxyRkatAlasR*. Strains null for *oxyR* and *katA* are defective for the defensive response to oxidative stress [36]; accordingly, the *oxyRkatA* mutant exhibited increased susceptibility to paraquat compared with wild-type (Fig. S1). However, the *oxyRkatAlasR* triple mutant was even more resistant to paraquat than was wild-type, confirming and extending the observation that a *lasR* mutation confers resistance to ROS (not shown). Similarly, the *oxyRkatA* mutant was more susceptible to tobramycin (Fig. 4E) (as shown for the *oxyR* single mutant and the aminoglycoside gentamicin [36]) and to ciprofloxacin (not shown) than was wild-type; as with paraquat, the *oxyRkatAlasR* triple mutant exhibited resistance to each of these drugs, an effect that was reversed by complementation with a wild-type copy of *lasR* on a plasmid (Fig. 4E and data not shown). These results indicate that *lasR* mutation confers resistance to these two antibiotics through its effects on respiratory activity and oxidative stress response. As *lasR* mutant strains and isolates also exhibit increased tolerance to some β-lactams due to increased β-lactamase activity [11], these results suggest that the emergence of *lasR* mutant isolates during chronic infections could adversely impact the clinical response to all three of the antibiotic classes used most commonly during standard CF treatment (β-lactams, fluoroquinolones, and aminoglycosides). The recent discovery [42] that increased bacterial production of NO· (which is increased by LasR inactivation, Fig. 1E) confers additional protection against a wide variety of antibiotics, including β-lactams, quinolones, and aminoglycosides, further supports this possibility.

## Discussion

In this work, *P. aeruginosa* isolates with inactivating mutations in the AHL-responsive transcriptional regulator LasR exhibited a profound growth advantage with nitrogen substrates found in the CF airway. These differences are attributable to *lasR*-dependent increased utilization of nitrogen oxides and decreased utilization of oxygen. This metabolic shift results in an increase in the production of the RNS NO·, and a corresponding decrease in the ROS O_2_^−^·, the latter of which is associated with decreased susceptibility in our conditions to at least two antibiotics used frequently in treating CF lung infections. This growth advantage in conditions characteristic of CF airways, and the resulting antibiotic resistance, may explain the observed high prevalence of LasR mutants and the associated worse lung function of CF patients whose airways contain these mutants [8].

**Figure 6:**
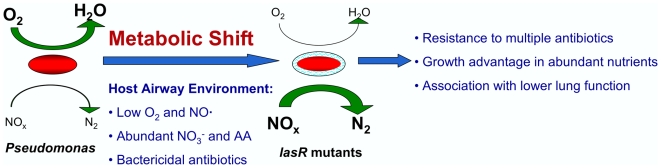
A model for metabolic changes in CF-adapted *lasR* mutant isolates of *P. aeruginosa*. According to the model, patients are initially infected with environmental isolates carrying wild-type copies of the *lasR* gene (left). These isolates have relatively high utilization of oxygen (activities indicated by the sizes of the green arrows) and lower utilization of nitrogen oxides (NO_x_). Selective pressures encountered in the host, including abundant host NO_3_^−^ and amino acids (AA), low host NO·, the presence of other bacterial species that metabolize NO·, reduced O_2_ concentrations, and treatment with β−lactams or antibiotics that generate ROS, favor the emergence of *lasR* mutant isolates with higher utilization of nitrates and lower utilization of oxygen. This metabolic shift confers a growth advantage in the nutrient conditions in the CF airway, including abundant NO_3_^−^, and relative resistance to the antibiotics used most frequently to treat CF patients.

The metabolic changes that occur upon *lasR* inactivation would be predicted to favor growth of *lasR* mutants arising spontaneously in the CF airway due to the confluence of selective forces encountered in this environment. For example, the abundant NO_3_^−^ and NO_2_^−^ [17],[18] and low oxygen tensions [20] found in CF secretions, as well as the relatively low NO· levels [18], would provide optimal metabolic conditions for *lasR* mutant selection. As suggested previously [43], *P. aeruginosa* likely adapts to a continuum of different oxygen tensions, with variation in the relative ratio of oxygen and nitrate utilization. Inactivating mutations in *lasR* may confer advantages in a variety of these microenvironments found in the CF lung. Also contributing to the beneficial nature of this environment for *lasR* mutant growth is the presence of NO·-detoxifying microbes, such as *S. aureus* and perhaps anaerobic bacteria, the latter of which were recently found to occupy CF secretions at high densities [44]; it should be noted that, while contact of *lasR* mutant *P. aeruginosa* with wild-type *P. aeruginosa* was also shown previously to reverse autolysis and sheen [11], it is not yet clear whether the mechanism of this effect is similar to that of *S. aureus*. The availability of amino acids as nutrient sources in CF secretions [13] would provide an additional selective pressure for *lasR* mutant growth [11]. Similarly, *lasR* mutants are relatively resistant to sources of oxidative stress, including tobramycin and ciprofloxacin (Figs. 4D–E), two antibiotics that, along with ceftazidime (to which *lasR* mutants are also relatively tolerant due to augmented β-lactamase activity [11]), are among the antibiotics used most commonly in CF treatment [41]. Although other sources of ROS are present in CF airways, such as H_2_O_2_ from host cells [36], whether exogenously adding these molecules to *P. aeruginosa* effectively confers intracellular oxidative stress is not as clear as is is the effect of the above antibiotics [22],[23]. While the results presented here demonstrate that nutrient conditions (particularly relating to oxygen and nitrogen oxides) are sufficient to enrich for *lasR* mutant growth *in vitro*, the frequent treatment of CF patients with the above antibiotics likely provides additional selection for these mutants, resulting in a complex dynamic between the CF airway nutrient environment, *P. aeruginosa* adaptation, therapy, and pathophysiology. These ideas are summarized in the model in Fig. 6.

There are multiple therapeutic and pathophysiologic implications of the model in Fig. 6. For example, assuming that *P. aeruginosa* infection leads to airway inflammation, and thus to obstructive lung disease, as suggested by current models of CF pathogenesis [41], the growth advantage of *lasR* mutant cells within the CF airway would be predicted to render such mutants more pathogenic to CF patients by virtue of higher cell density and greater consequent inflammation. (It should be noted that while *lasR* mutant *P. aeruginosa* strains were shown to be less pathogenic in animal models of short-term respiratory infection [45], those models may not accurately reflect the pathogenic mechanisms of chronic CF airway infection, during which many “acute” virulence factors are not expressed [46]). This effect may contribute to the observed association between *lasR* mutant CF airway infection and worse lung function [8]. Furthermore, the clinical response to standard antibiotic therapy in patients infected with *lasR* mutants would be predicted to be poor relative to patients with wild type isolates, perhaps further contributing to the clinical impact and rendering eradication increasingly difficult [8]. Thus, the presence of *lasR* mutants in CF respiratory cultures may be of prognostic value, and aggressive, directed treatment of these mutants upon isolation (i.e., through the expanded use of monobactams, tetracyclines, or polymyxin in the case of *lasR* mutant infection) or with regimens that do not select for their growth may lead to improved outcomes.

While recent publications have shown that quorum sensing regulates the expression of denitrification genes [14],[15],[16] and oxygen metabolic genes [30] at the transcriptional level, the mechanism of the distinct metabolic behaviors of *lasR* mutant and wild-type cells is likely to be as complex as the quorum sensing system itself. In *P. aeruginosa*, quorum sensing involves at least three parallel signaling systems, at least four different signal receptors, and regulation by diverse environmental cues [14],[47],[48]. However, some mechanistic clues are evident from our results. Previously, we showed that the two-component metabolic regulatory system CbrAB contributes to the metabolic phenotypes of *lasR* mutant clinical isolates of *P. aeruginosa* [11]; mutants in this system have decreased capacities to use amino acids as nitrogen sources [49], and *lasR* mutant isolates have upregulated expression of the transcriptional metabolism regulator *cbrB* [11]. The current results also suggest an additional mechanism for the growth advantage of *lasR* mutant *P. aeruginosa* in specific amino acids (most markedly with phenylalanine, but also with other aromatic and branched-chain amino acids [11]). Many enzymes that metabolize amino acids are inactivated by reactive oxygen species (ROS), including the first enzyme in the phenylalanine catabolic pathway, phenylalanine hydroxylase [50],[51]. Therefore, cells with lower intracellular concentrations of ROS, such as *lasR* mutants (Fig. 4B), would be predicted to be better able to utilize amino acids such as phenylalanine as nutrient sources. Additionally, the *las* system is involved in regulating the levels and timing of production of a family of hydroxyalkylquinoline (HAQ) molecules [52], including the compounds 4-hydroxy-2-heptylquinoline (HHQ), the overproduction of which generates the sheen characteristic of *lasR* mutant colonies [11]; its N-oxide HQNO, which is a redox-cycling agent [29]; and the *Pseudomonas* quinolone signal (PQS) [52]. Exposure to PQS was shown to modify *P. aeruginosa* responses to reactive oxygen species and ciprofloxacin [38], suggesting a functional linkage between HAQs, oxidative stress responses, and susceptibility to fluoroquinolones. Therefore, these quinolines may regulate metabolic properties in both source and neighboring cells, and temporal differences in their production resulting from LasR inactivation may contribute to the observed metabolic changes.

Numerous explanations have been offered for the identification of *lasR* mutant *P. aeruginosa* in diverse clinical and experimental conditions [10],[11],[12],[53],[54],[55],[56],[57],[58],[59],[60]. For example, in experimental growth medium in which *P. aeruginosa* growth requires the production of *lasR-*regulated protease, *lasR* mutants emerge that “cheat” from the protease produced by wild-type strains [59],[60]. However, CF sputum is abundant in free amino acids [13] (upon which *P. aeruginosa lasR* mutants can grow without requiring protease [11],[59]), and it has been shown that both laboratory strains [61] and clinical isolates [12] may produce protease in the absence of a functional *las* system. Furthermore, *lasR* mutants are frequently isolated from CF sputum without detectable wild-type co-isolates [2],[11]. These findings suggest that cheating alone does not explain the high prevalence of *lasR* mutants among people with CF. Alternatively, it has been suggested that *lasR* mutants emerge due to physiological characteristics that confer relative fitness advantages in specific growth conditions [11],[56]. The current results support the hypothesis that *lasR* mutant *P. aeruginosa* have a growth advantage in nutrient and antibiotic conditions found in the CF airway (as summarized in Fig. 6). While it is unclear which of these forces, antibiotics or nutrients, predominates *in vivo* in selecting for inactivating *lasR* mutations, their combination would be predicted to exert powerful pressure against LasR function.

The hypothesis that *P. aeruginosa* adaptation to the CF airway is driven in large part by metabolic forces found in CF airway secretions is supported by findings with other adapted mutants from chronic infections. For example, mucoid *P. aeruginosa* strains and isolates also exhibit upregulated NO_3_^−^ metabolism relative to non-mucoid *P. aeruginosa* and, as a result, are more susceptible than nonmucoid isolates to acidified NO_2_^−^ [27]. Furthermore, the mucoid phenotype is promoted by hypoxia [20]. Similarly, *P. aeruginosa* isolates with another CF adaptation, mutations that upregulate the glucose-6-phosphate dehydrogenase gene *zwf*, confer resistance to oxidative stress and paraquat [62],[63]. The enrichment for *lasR* mutant *P. aeruginosa*, with growth advantages in CF airway conditions, by *S. aureus* is also reminiscent of the reverse interaction: the selection of *S. aureus* metabolic mutants, known as small-colony variants (SCVs), due to co-culture with wild-type *P. aeruginosa* [29]. *S. aureus* SCVs are defective for aerobic growth, are resistant to aminoglycoside antibiotics such as tobramycin, and frequently exhibit both increased expression of denitrification genes [64] and associated increased susceptibility to NO_2_^−^ [65], much like *lasR* mutant *P. aeruginosa*. The symmetry of this *S. aureus-P. aeruginosa* relationship, in each direction favoring the growth of antibiotic-resistant, metabolic mutants with decreased aerobic activity, further suggests a common mechanism for selection during chronic CF infections, and perhaps during many other chronic infections, driven by the metabolic forces present in host tissues. In support of this hypothesis, the likelihood of persistent, latent infection by the respiratory pathogen *Mycobacterium tuberculosis* is thought to be determined in large part by the lung metabolic milieu, particularly the relative ambient concentrations of nitrogen and oxygen species [66]. Similarly, the pathogenic fungus *Cryptococcus neoformans* exhibits early metabolic adaptations in animal models of chronic pulmonary infection, including altered responses to nitrosative stress and superoxide [67]. As with *M. tuberculosis* [68], these findings support the concept that chronic CF airway infections with *P. aeruginosa* could be amenable to therapies that increase airway nitrosative stress. Such therapies could include inhaled NO_2_^−^ [27] or L-arginine [19], two treatments already being examined as candidate CF treatments. Our results support the utility of these treatments both in preventing *P. aeruginosa* adaptive changes associated with advanced lung function decline [7],[8] and that may be attributable to current antibiotic regimens (Fig. 6), as well as in treating patients with advanced infection in which these adaptations have already occurred.

In summary, the nutrient conditions characteristic of the CF airway select for growth of *lasR* mutant *P. aeruginosa*, resulting in decreased susceptibility to antibiotics without the need for antibiotic exposure. Adaptation of many microbes to new environments during chronic infections may commonly result in metabolic changes that impact response to antibiotics. This scenario may be particularly relevant for opportunistic pathogens such as *P. aeruginosa*, many of which naturally occupy competitive and nutrient-poor environmental niches like soil and water, as they adapt to the specific nutrient conditions found in host environments such as the nitrogen-rich CF airway.

## Materials and Methods

### Bacteria

Table 1 lists the bacterial strains and isolates used in this work, except for the strains carrying transposon insertion mutations in nitrate metabolic genes, which were obtained from the PA14 transposon insertion mutant library [69]. The origins of all strains and isolates are described in the references provided in Table 1, except for the *narK1K2* and *narK1K2lasR* mutants, described below.

**Table 1:** List of strains used in the described experiments.

Strain name	Description	References
***P. aeruginosa***		
PA14	Laboratory strain with phenotypic and genotypic features that resemble many clinical isolates	[11]
PA14-*lasR*::Gm	Engineered mutant of PA14 with a gentamicin resistance cassette inserted into the *lasR* gene	[11]
PA14-L1	Spontaneous *lasR* mutant of PA14 without an antibiotic resistance marker	[11]
PA14Δ*lasR*	PA14 with an unmarked deletion in *lasR*	This study
AMT0023-30	CF clinical isolate with wild-type *lasR* allele from a young patient (previously referred to as Patient 1 Early)	[2],[11]
AMT0023-30-L1	Spontaneous *lasR* mutant of AMT0023-30	[11]
AMT0023-94	CF clinical isolate from the same patient as AMT0023-30 but 8 years later with a naturally-occurring *lasR* mutation (previously referred to as Patient 1 Late)	[2],[11]
NC-AMT0101-3	CF clinical isolate with wild-type *lasR* allele from a second young patient	[2],[11]
NC-AMT0101-2	CF clinical isolate collected from the same patient as NC-AMT0101-3 but 8.6 years later with wild-type *lasR* allele and mucoid phenotype	[2],[11]
NC-AMT0101-1	CF clinical isolate from the same culture as NC-AMT0101-2 with naturally-occurring mutant *lasR* allele, nonmucoid.	[2],[11]
PAO1	Laboratory strain	[36]
*oxyR*	PAO1 with unmarked deletion in *oxyR*	[36]
*oxyRkatA*	Above *oxyR* mutant with *katA*::Gm	[36]
*oxyRkatAlasR*	*lasR* mutant that emerged spontaneously during growth for 3 days on LB agar from *oxyRkatA* with the mutation G191V	This study
*narK1narK2±lasR*	Strains with clean deletions in *narK1* and *narK2* with and without clean deletions in *lasR*	This study
***S. aureus***		
*S. aureus* Newman strain	Laboratory strain of *S. aureus*	[24]
*hmp*	Derived mutant *S. aureus* Newman with the gene that encodes the NO* detoxifying flavohemoprotein deleted	[24]
AMT0064-6	Clinical CF isolate of *S. aureus*	[29]

### Mutant construction and plasmids

Each deletion in the *lasR*, *narK1K2* and *narK1K2lasR* mutants was generated using allelic exchange with *sacB-*containing counterselectable gene replacement vectors using sucrose counterselection essentially as described [70]. Briefly, the *lasR* gene was entirely deleted from the chromosome except for the start and stop codon, using the plasmid *sacB*-based pEX18Gm for integration and excision. The *narK1-narK2* genes, which are organized tandemly as an operon, were deleted as a one continuous stretch of DNA using identical methods, both in wild-type PA14 as well as the *lasR* mutant background. The deletion removed the *narK* coding sequence beginning from the 30^th^ codon of *narK1* until the 462^nd^ codon of *narK2*, leaving the first 29 codons of *narK1* and the last 7 codons of *narK2* intact.

The plasmid pUCPSK-*lasR* was the kind gift of Eric Déziel and was used for complementation of *lasR* deficient strains and isolates as described [61].

### Growth conditions and chemicals

Except where indicated, all cultures were inoculated from LB overnight cultures of bacteria or cells suspended from LB agar cultures. Liquid static cultures were grown in LB with 400 µM KNO_3_ (Sigma) except where indicated otherwise. Phosphate buffered LB agar was prepared as described [27]. Chemically defined PN medium was prepared as previously described [24], and consists of a phosphate buffer supplemented with a carbon source (glucose), nitrogen and sulfur sources [(NH_4_)_2_SO_4_ and MgSO_4_], amino acids, nucleic acid bases, and vitamins (thiamine, niacin, biotin, and pantothenic acid).

### Chemicals

Hemoglobin, hydroethidine, tobramycin, paraquat (methylviologen dichloride hydrate), potassium nitrate, and sodium nitrite were obtained from Sigma. NO donors DEANO (DEA-NONOate) and ProliNO (Proli-NONOate) were purchased from AG Scientific (San Diego, CA) and SperNO was obtained from CalBiochem (San Diego, CA). Ciprofloxacin was from Biochemika/Sigma. Prepared antibiotic disks with tobramycin, kanamycin, gentamicin, carbenicillin, tetracycline, aztreonam, ceftazidime, and polymyxin B were from Becton Dickinson. Growth media and agar were from Becton Dickinson & Co.

### Growth assays

Growth of cells in the indicated liquid media was measured optically using a BioScreen C Microbiology Microplate reader (Growth Curves USA, Piscataway, NJ) without shaking (except immediately prior to readings), a condition that limits oxygen mass-transfer. Assays to look for mutant sectors were performed by inoculating 10 µl drops of 1∶10-diluted overnight cultures on LB with 400 µM KNO_3_, followed by incubation at 37°C for 24 hours and then at room temperature for up to approximately 1 month thereafter.

### Assays for denitrification activity and NO_2_^−^ susceptibility

NO· was quantified using an ISO-NOPMC Mark II electrode (WPI Instruments, Fl) and dissolved oxygen was measured in parallel using a Clark-type electrode MLT1120 (ADI Instruments) with standard curves as per manufacturer instruction. Data from both probes were analyzed through an Analog Adapter MLT1122 (ADI Instruments). NO_2_^−^ disk diffusion on acidified, buffered LB agar was performed as described [27], except that all incubations were performed with aerobic growth.

### Assay for oxygen utilization

Respiration rates in liquid cultures were measured by resuspending PBS-washed cells in prewarmed LB with 400 µM KNO_3_ in a microrespiration system (Unisense AS, Denmark). Calibrations were performed according to manufacturer's instructions using air-purged and argon-purged growth medium.

### Hydroethidine assay

Fluorescence after hydroethidine addition to lawns of cells during growth on LB agar (similar results were obtained with and without added NO_3_^−^) was measured using excitation/emission wavelengths of 396/570 nm [32], followed by photography and quantitation using NIH ImageJ software (NIH, Bethesda, Md, http://rsb.info.nih.gov/ij/).

### Agar growth assay

Agar-suspended cultures were grown in 0.9% LB agar inoculated with equal cell numbers of all cell types- approximately 10^5^ CFU of the indicated strains (resulting in a final cell density of approximately 2×10^3^ CFU/mL), except when indicated otherwise- and with chemicals and antibiotics added as indicated. In each case, the prepared agar was inoculated with bacteria when the medium had cooled after autoclaving to approximately 37°C but before gelling. The medium was then poured into 10 mL syringes from which the port ends had been removed, leaving an open end, which was loosely covered for incubation. After incubation, the plunger of the syringe was depressed slowly, ejecting a cylinder of culture. Serial, 1.5 mm slices of culture were removed and added to 1 mL each of sterile PBS, and vortexed for 30 seconds before enumeration of cells from the resulting solution by plating.

### Nitrogen metabolic assays

NO_2_^−^ production was measured using the Griess Reagent System kit (Promega, Madison WI). Nitrate was quantified enzymatically using a commercially available reagent set (R-Biopharm, Marshall, MI). Rates of NO· degradation were determined as previously described [24]; briefly, five milliliter cultures in PN medium were grown by shaking at 37°C to an OD660≈0.4. Cells were then resuspended to 1×10^8^ cfu ml−1 in 8 ml final volume. A two-hole rubber stopper sealed with Parafilm enclosed the cell suspension in an 8 ml glass vial with no gaseous headspace. Cells were stirred vigorously at 37°C as ProliNO was added through one open port to 1 µM. The resulting immediate release of approximately 2 µM NO· followed by the gradual decay of detectible signal was recorded and normalized to the fraction of initial [NO·]. Measurements were performed in triplicate for each strain tested. NO· susceptibility was determined by measuring the lag in growth after bacterial cultures in LB medium were supplemented with 0.5 or 2.5 mM SperNO (t_1/2_ = 39 min at 37°C).

### Oxygen metabolism in deep-agar cultures

Deep-agar cultures inoculated with serial dilutions of *P. aeruginosa lasR* and wild-type cells in LB-0.9% agar with and without 400 µM KNO_3_ and with and without paraquat were grown overnight at 37°C. Oxygen concentrations were subsequently recorded using a microsensor setup (OX 10 oxygen microsensor, PA 2000 picoammeter, both from Unisense AS, Denmark) at 37°C in a preconditioned water bath. Data were recorded using SensorTrace Basic software (Unisense). The probe was advanced into the agar, and measurements taken, in 50 µm increments.

### Statistics

Differences between experimental measurements were computed using unpaired, two-tailed Student's t-tests.

## Supporting Information

Figure S1 *lasR* inactivating mutation decreases susceptibility to paraquat. Experiment performed as described in Fig. 4C, except with the indicated strains. Results shown are averages ±s.d. for three replicates and are representative of two separate experiments.
(0.13 MB TIF)
