# Supplementary figures and images for: Nutrient Availability as a Mechanism for Selection of Antibiotic Tolerant Pseudomonas aeruginosa within the CF Airway
Source: PLoS Pathog. 2010 Jan 8;6(1):e1000712. doi: 10.1371/journal.ppat.1000712 (PMC2795201; doi:10.1371/journal.ppat.1000712)

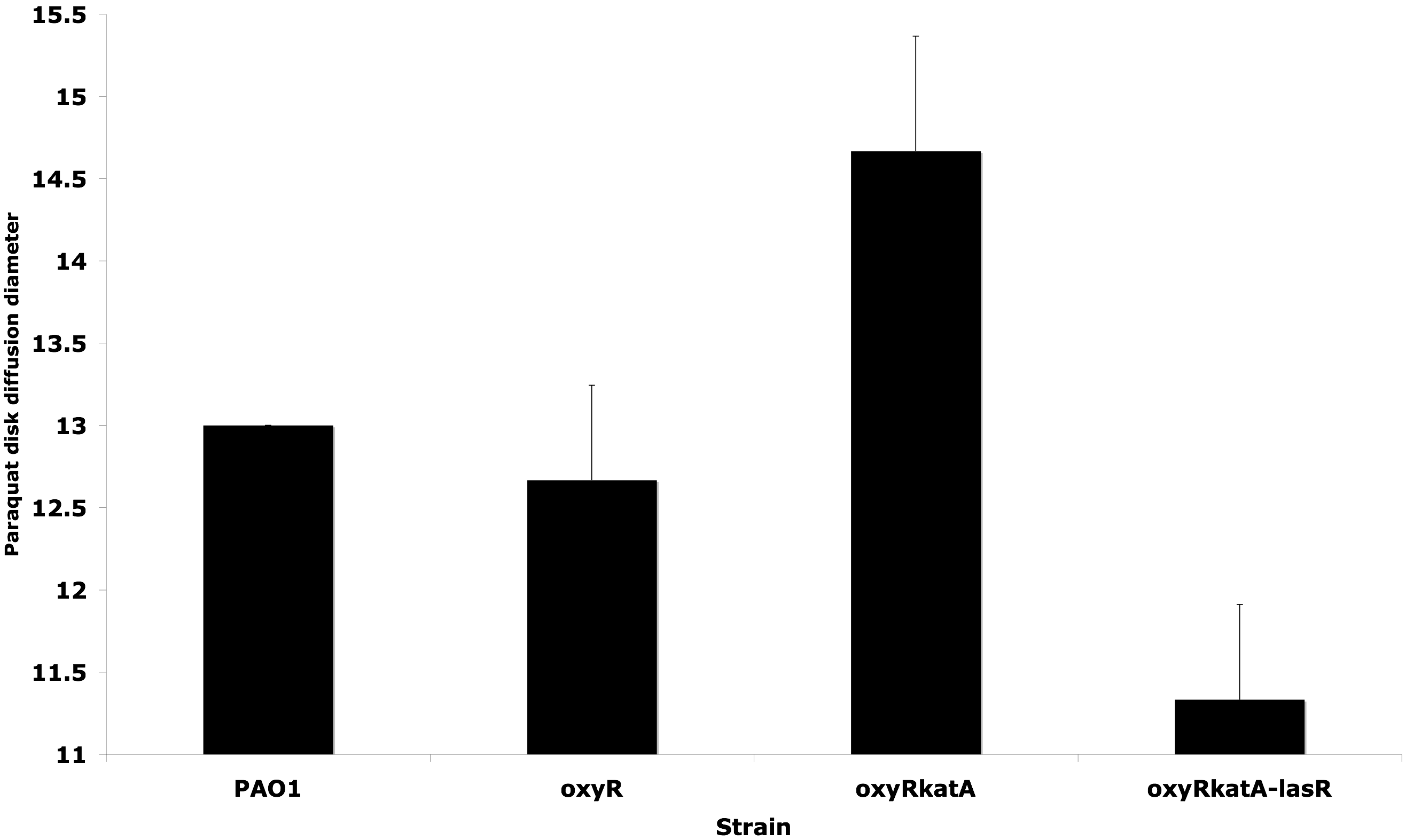

Supplement: Figure S1 — lasR inactivating mutation decreases susceptibility to paraquat. Experiment performed as described in Fig. 4C, except with the indicated strains. Results shown are averages ±s.d. for three replicates and are representative of two separate experiments. (0.13 MB TIF) [file ppat.1000712.s001.tif]
